# Creating connections – the development of a mobile-health monitoring system for heart failure: Qualitative findings from a usability cohort study

**DOI:** 10.1177/2055207616671461

**Published:** 2016-10-10

**Authors:** Tracey Chantler, Chris Paton, Carmelo Velardo, Andreas Triantafyllidis, Syed A Shah, Emma Stoppani, Nathalie Conrad, Ray Fitzpatrick, Lionel Tarassenko, Kazem Rahimi

**Affiliations:** 1 George Institute for Global Health, University of Oxford, UK; 2 London School of Hygiene and Tropical Medicine, UK; 3 Institute of Biomedical Engineering, University of Oxford, UK; 4 Health Services Research Unit, University of Oxford, UK; 5 Division of Cardiovascular Medicine, University of Oxford, UK

**Keywords:** Qualitative research, heart failure, digital health, mobile health, self-care, patient participation

## Abstract

**Objective:**

There is significant interest in the role of digital health technology in enabling optimal monitoring of heart failure patients. To harness this potential, it is vital to account for users’ capacity and preferences in the development of technological solutions. We adopted an iterative approach focussed on learning from users’ interactions with a mobile-health monitoring system.

**Methods:**

We used a participatory mixed methods research approach to develop and evaluate a mobile-health monitoring system. Fifty-eight heart failure patients were recruited from three health care settings in the UK and provided with Internet-enabled tablet computers that were wirelessly linked to sensor devices for blood pressure, heart rate and weight monitoring. One to two home visits were conducted with a subgroup of 29 participants to evaluate the usability of the system over a median follow-up period of six months. The thematic analysis of observational data and 45 interviews was informed by the domestication of technology theory.

**Results:**

Our findings indicate that digital health technologies need to create and extend connections with health professionals, be incorporated into users’ daily routines, and be personalised according to users’ technological competencies and interest in assuming a proactive or more passive role in monitoring their condition.

**Conclusions:**

Users' patterns of engagement with health technology changes over time and varies according to their need and capacity to use the technology. Incorporating diverse user experiences in the development and maintenance of mobile-health systems is likely to increase the extent of successful uptake and impacts on outcomes for patients and providers.

## Introduction

Heart failure (HF) represents a significant burden to patients and health services globally.^1–3^ Despite improvements to the treatment and management of HF, death and readmission after discharge remain high.^1^ Constraints in the delivery of health care services are likely to be a contributing factor to these poor outcomes. Recent research suggests that health-care professionals perceive disease management for this multi-morbid patient population as complex and unpredictable, and do not have sufficient time or human resources to achieve optimal monitoring and follow-up.^4^ Similarly, a qualitative systematic review concurred that difficulties in accessing health-care services present a significant challenge for patients, and identified social isolation, living in fear and losing a sense of control as the prominent features of living with HF.^5^

Technology enabled home-monitoring systems could help address gaps in health-care provision. The evidence concerning the clinical effectiveness, cost-effectiveness and sustainability of such systems in the HF population is however inconsistent and requires further investigation.^6–10^ Despite this uncertainty, qualitative research has indicated that HF patients who used a mobile phone-based system for six months felt more aware of their condition, less anxious and more empowered.^11^ Similarly, users of a routine telemonitoring service gained significant reassurance from practitioner surveillance.^12^ Elsewhere, however, concerns have been expressed about telemonitoring undermining positive ageing and self-reliance.^13^

Telemonitoring systems require end-users to change their behaviour by adopting technology to monitor their condition, however the need to tailor such systems to user’s capacity and preferences is not always given sufficient priority.^14,15^ The Medical Research Council (MRC) recommends that end-users should be involved in assessing the acceptability and usability of a behavioural intervention before scaling it up for further clinical evaluation.^16^ We applied these recommendations to develop and evaluate a home-monitoring system with good usability for HF patients. This programme of work includes several steps and this article describes the first step and is referred to as SUPPORT HF (Seamless User-centred Proactive Provision Of Risk-stratified Treatment for Heart Failure) 1. The multidisciplinary research team included biomedical engineers (CV, AT, SS), a cardiologist (KR) and a clinician with expertise in health informatics (CP), a research nurse (ES), a medical sociologist (RF) and a qualitative researcher (TC). In this article we report on the qualitative component of our study, which documented participants’ interactions with the system in order to evaluate its usability, explore how they incorporated self-monitoring activities into their daily routines, and determine what functions the system played in their lives.

## Methods

### Design

SUPPORT HF1 was a mixed methods non-interventional cohort study, which aimed to develop and evaluate the usability of a mobile-health (mhealth) home-monitoring system for HF patients with varying degrees of physical and cognitive functioning. To achieve this we applied a usability framework^17^ and adopted an iterative and participatory approach informed by action research^18^ and agile software development.^19^ In the initial development phase, we held a co-design workshop with 15 HF patients. This increased our understanding of patients’ requirements and capacity for home monitoring, and informed decisions about hardware selection and software design. Subsequently a larger group of HF patients and their caregivers was involved in testing and adapting the SUPPORT HF mhealth monitoring system over an average period of six months. Our primary outcome measure was system usability, defined as ‘the extent to which a product can be used by specified users to achieve specified goals with effectiveness, efficiency, and satisfaction in a specified context of use’.^17^ Qualitative and quantitative methods were employed to identify, understand and iteratively address usability issues. In this paper we focus on the qualitative findings, the quantitative results are reported in a parallel paper.^20^

The monitoring system recorded user interactions (e.g. frequency and duration of system usage) and transmitted these securely via 3G/4G Internet connections to a web-server hosted within the National Health Service (NHS) network infrastructure. This quantitative data was combined with information gained from the home visits (observations and interviews), and used to adapt and improve the usability of the system.

### Participants, sampling and recruitment

**Figure 1.**
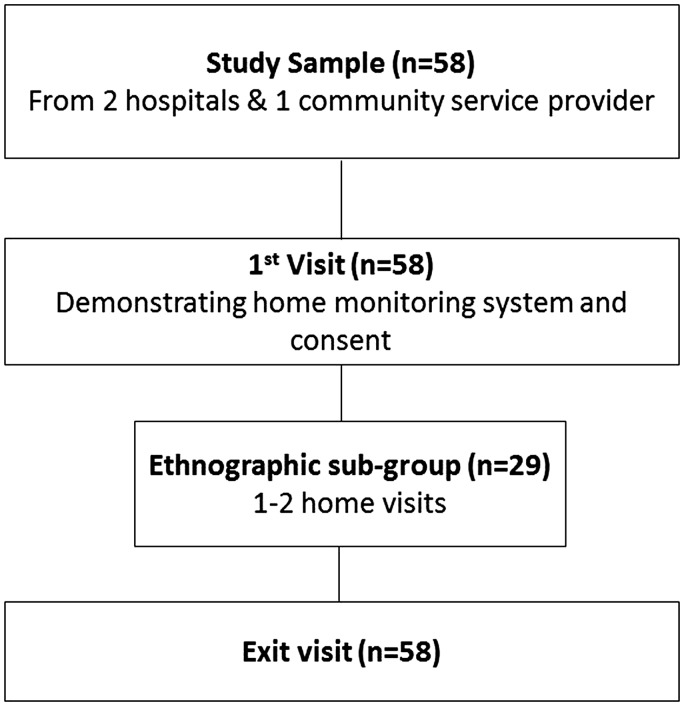
Seamless User-centred Proactive Provision Of Risk-stratified Treatment for Heart Failure (SUPPORT HF 1) participant flow diagram.

The study involved 58 HF patients recruited from two hospitals (acute and ambulatory settings) and one community HF service provider in South Central England (Figure 1). Patients aged >18, suffering from any severity of HF, with good command of written and spoken English were eligible to participate. Purposive maximum variation sampling was used to achieve a comprehensive sample in terms of gender, age, ethnicity, demographic and clinical characteristics (e.g. age >75, multi-morbidities) and social circumstances. Patients, who expressed interest in the study were contacted and visited by CP or ES to explain the purpose of the study and what it involves and to show them how to use the mhealth system. They were advised that participation in the study was complementary to routine health care provision, meaning that the researcher team would not assume responsibility for medical management, but would advise participants to contact their general practitioner (GP) when necessary. Informed consent was obtained from patients who decided to enrol in the study. Clinical and socio-demographic data were then collected and depending of their date of enrolment, participants were asked to use the mhealth system for a period of 3–12 months. The first participant was recruited in June 2013, the last in May 2014 and the median follow-up was six months (interquartile range (IQR) 3.6–9.2). The relatively wide and flexible follow-up duration served two purposes: (a) to determine the duration of home monitoring that is acceptable for patients, (b) to evaluate the usability of iterative modifications introduced to system following feedback from initial participants, by asking these participants if the modifications improved the system, and gaining feedback from new participants.

### The mhealth home-monitoring system

The mhealth system and its development are reported in more detail in a parallel paper.^21^ In short, the system consisted of a mobile application installed on a touch screen Android-based tablet computer connected to a blood pressure monitor and a set of weighing scales using the Bluetooth wireless communication standard. Participants were asked to complete symptom diaries and take measurements on a daily or regular basis, and complete quality of life questionnaires every three months. These data were transmitted to a back-end infrastructure through the Internet, enabling the study team to review the participants’ health status remotely. In-built alerts were issued if a participant’s measurements indicated any deterioration in their condition that required them to contact their general practitioner (GP), and the study team could also send text messages to comment on participants’ health status. The application also includes features that allow participants to review their personal readings via a graphical display, access educational materials and communicate with the study team by pressing a ‘contact me’ button in case of any usability problems.

### Qualitative study component

The purpose of the qualitative component of this study was to document participants’ interactions with the mhealth system in order to evaluate its usability, explore how they incorporated self-monitoring activities into their daily routines and determine what functions the system played in their lives. We adopted an ethnographic approach for this part of our study which is one of the approaches recommended in Daniels et al.’s usability framework.^17^ In order to achieve our purpose, we conducted a series of 1–2 home visits (HVs) with a subgroup of 29 participants and an exit visit with all participants who completed the study. These HVs comprised of observations of participants’ use of the system, and semi-structured interviews which were conducted by a researcher (TC) with expertise in qualitative research methods. The subgroup included the first 21 participants (two HVs) and eight additional participants (one HV), who were selected to ensure that this group was suitably diverse. The decision to limit the size of this subgroup to 29, and to undertake one or two HVs, was informed by theoretical saturation i.e. the point at which no new concepts emerge from the review of successive data collection.^22^

The first HV occurred within four weeks of participants joining the study and the second 3-4 months into the study. Observations during the first visit focused mainly on practical usability, and the interviews explored participants’ illness narratives, coping strategies and social context, their interest in the system and motivations for using it, and their initial experience of adopting this health technology. The focus of the second HV was to explore how participants were incorporating the use of the system and related self-monitoring activities into their daily lives. This involved observing and recording in field notes how and where the system was used and placed, asking participants to describe their patterns and experiences of use (what they find difficult or easy and why), and exploring their views about the usefulness of the system and related self-monitoring activities. The exit home visit repeated some of the latter and included a debriefing process.

In total 29 first and 16 second HVs were conducted with subgroup participants and 48 exit visits with all participants, apart from two who were not available, four who had died during the study, and four who had withdrawn. Of the four early withdrawals, three were visited and interviewed after their decision to withdraw. In addition, TC responded to the please ‘contact me’ requests issued by participants regarding usability related questions.

### Qualitative data analysis

The qualitative data comprised of 45 interviews and field notes compiled as part of HVs, early withdrawal/exit visits and ‘contact me’ follow-up calls. Interviews were audio-recorded, transcribed and imported with the field notes into a qualitative data analysis software programme (NVivo 10). Our analysis was iterative and involved TC reading the transcripts and field notes and listening to the audio-recordings and considering how the observational data and interview data could be accounted for intelligently. After completing an initial thematic analysis^23^ of data from the first five home visits, key investigators agreed it would be useful to add a theoretical angle to our analysis. We identified a theory that matched our purpose and decided to use this to inform our data analysis.

The domestication of technology theory^24,25^ is defined as:

The domestication of technology concept is used to describe and analyse the processes of acceptance, rejection and use. Users are seen as social entities and the model aims to provide a framework for understanding how technology innovations change, and are changed, by their social contexts.^26^

Four dimensions of domestication are described: appropriation, objectification, incorporation and conversion. Appropriation addresses questions such as why were participants interested in taking part in the study and using the system, and what motivated them. Objectification is about determining what the mhealth system will be used for. Incorporation is about practical hands-on use and we extended it with an additional construct to convey how participants continued to evaluate the usefulness of the system as it became more or less integral to their daily lives. Lastly, conversion deals with expressed aspirations and continuing interest in using the system.

These dimensions were reflected in the interview topic guides, and in the individual case studies that were developed to elucidate and compare the process of domestication across the ethnographic subgroup. Essentially, we applied these dimensions as analytical constructs with some minor modifications to reflect our subject matter. TC led the analysis and reported back to the multi-disciplinary research team at regular team meetings in order to increase the transparency of the analysis and verify and crosscheck findings with the quantitative data. This process added rigour by providing a forum for interrogating analytical decision-making, identifying areas that needed further examination and ensuring that the analysis was conducted in a systematic and comprehensive manner.

## Results

### Participants

The main study sample (Table 1) included 58 people with a median age of 77 years (range 21–94), 21 (36%) were female, three (5%) represented ethnic minorities, 40 (23%) suffered from diabetes and 10 (18%) from Chronic Obstructive Pulmonary Disease (COPD). From a social perspective 12 (21%) participants lived on their own, three (5%) lived in sheltered accommodation, 39 (67%) lived with their partner, and four (7%) resided with other family members. The qualitative research subgroup did not differ considerably from the main sample apart from the fact that it included all participants from ethnic minority backgrounds and less people living on their own (Table 1).

**Table 1.** Baseline socio-demographic and clinical characteristics.

	All participants (*n* = 58)^a^	Ethnographic subgroup (*n* = 29)
Age in years, median (IQR)	77 (64–82)	74 (62–82)
Women, *n* (%)	21 (36)	10 (34)
Ethnic minorities, *n* (%)	3 (5)	3 (10)
Diabetes mellitus, *n* (%)	23 (40)	13 (45)
Chronic obstructive lung disease, *n* (%)	10 (18)	6 (21)
Level of competency in use of digital technologies		
Very limited or none, *n* (%)	27 (48)	13 (45)
Competent, *n* (%)	23 (41)	14 (48)
Expert, *n* (%)	6 (11)	2 (7)
Living arrangement		
With partner, *n* (%)	39 (67)	21 (72)
With other family members, *n* (%)	4 (7)	3 (10)
Alone, *n* (%)	12 (21)	4 (14)
Sheltered accommodation, *n* (%)	3 (5)	1 (3)
Self-assessed severity of symptoms		
NYHA class 1, *n* (%)	9 (16)	3 (11)
NYHA class 2, *n* (%)	25 (45)	12 (44)
NYHA class 3, *n* (%)	16 (29)	10 (37)
NYHA class 4, *n* (%)	6 (11)	2 (7)

IQR: interquartile range; NYHA: New York Heart Association.

a Level of competency and symptom assessment could not be assessed in two patients who withdrew before using the system.

### Appropriation

Under the term appropriation, we were interested in understanding ‘why’ participants chose to help develop a user-friendly mhealth system to monitor health-related variables. This included gaining an understanding of their motivations, hesitations and any other influencing factors.

Altruistic sentiments were the dominant motivational force for taking part in this study. Participants expressed a desire to help others and give something back to the health system with which some of them described an intense relationship. Two participants (P3, P36) expanded on this idea of reciprocation by detailing their interest in the future role of digital technology in medicine.

A female participant (P22) attributed a psychological benefit to participation describing how it replaced the charity work she could no longer do. Similarly, when asked about how he benefited from using the system a male participant explained: ‘I feel that I am giving something back…. it is the little children in hospital that kill me – it’s for them that I am doing this’ (P38).

Initial hesitations about taking part in the study included: (a) feeling undeserving of the system and associated costs (P28), (b) reservations about removing the system and perceived support at the end of the study (P18), and (c) apprehensions about using new technology. ‘I’ll have a go yeah, but then I thought to myself “Oh blow you” because I weren’t brought up in pushing buttons and upper wires I calls it’ (P16).

Despite being informed at the beginning of the study that the system was not being used to actively monitor the patients and was only being assessed for usability and development purposes, a recurring theme in their considerations about the benefits of participating and using the system was the concept of a ‘safety net’ (P1). Knowing that ‘there is somebody out there who was picking up that these signs that you weren’t feeling well’ (P36) was described as a ‘bonus’ (P3) that ‘makes you more easy in your mind’ (P2). The external medical review enabled by use of the system conveyed a sense of connection, which in turn instilled confidence, reassurance and in some cases increased individual’s determination to manage their illness.

One of the most important things is confidence and in between the visits to the cardiologist or tests or test results or whatever you just trip along and you’re thinking, ‘Am I OK, am I… is it getting worse, am I… what was that?’ you know you just question everything. So you kind of like analyse everything about your body all the time. So having this [um] is just gold dust, it’s just reassurance and giving you the confidence, and knowing that yeah OK you’re doing all you can, but there’s somebody just kind of overseeing if anything drastic happens they can step in and it’s… and if you can keep your confidence about your health and then you’re going to be more determined I think to put your own fight in and lead a healthy and fitter life. So yeah it’s gold dust. (P1)

Participants’ social experiences of being diagnosed and living with HF were also of relevance to their decision to use the system. Diagnostic and prognostic uncertainties characterised these experiences, and some participants’ recounted difficulties in transitioning from hospital to community care. One man described how after a lengthy period in hospital he was ‘suddenly alone at home and scared to move’ (P6)*.* Learning how to manage a chronic illness, negotiate uncertainty and regain some control over their lives was described as challenging. Within this context the system was appropriated as a tool that may provide access to additional support.

### Objectification

Under the term objectification, we were interested in the ‘what for’ aspect of participants’ interaction with the mhealth system. This included understanding what participants use the system for, what does it represent for them, and what function does it play in their lives.

Our data indicated that the system represented an extension of care for participants; it connected them with health professionals thereby providing what they perceived to be a safe platform for practicing self-monitoring. Particular emphasis was placed on how the system helped them to become more self-sufficient. A participant in her early twenties thought the system was:

A really good idea… it kind of gives you some control over everything whereas when you get diagnosed with something like this you kind of feel like it’s out of your hands… it’s actually really good knowing that you have a little bit of control over your own management. (P21)

Similarly, a participant in her nineties described how she wanted to take part in order ‘to do something proactive about her condition’ (P26).

Two people thought that use of the system ‘may mean I don’t need to go to the hospital as much’ (P8), or ‘won’t have to access health services as much’ (P29). Expanding on this another person said that using the system

Makes me much more knowledgeable as to what’s going on, if something is not quite right, I wait normally for a day or so to see if it passes over, if it doesn’t I go and see my GP. So basically I’m covered. (P18)

For another participant (P19) the knowledge gained from self-monitoring translated into reassurance, making him more aware of his symptoms and giving him more control.

### Incorporation

Under incorporation we aimed to understand the particulars of ‘how’ participants interact with the system. This included documenting participants’ experiences of adopting and interacting with the system and making it part of their regular routines. The process of incorporation was influenced by participant’s competence in using digital technologies. Hence we devised three categories of user: (a) novice – those who have limited or no experience of using mobile phones and computers; (b) competent – those who use these technologies for personal, work or leisure activities; and (c) expert – those whose professional or voluntary activities require them to guide or teach others to use digital technologies. Based on this classification, 13 (45%) of the subgroup participants were novice, 14 (48%) competent and 2 (7%) expert technology users (for two participants this information was unavailable) (Table 1). This classification mainly helped us to look in more detail at the experiences of novice users, drawing attention to the process of gradual familiarisation and the need to integrate additional support functions to increase the level of novice user’s interactions with the system.

### Adopting and interacting with the system

Expert and competent technology users adopted the system very easily whereas the learning process for novice users involved a comparatively steep learning curve and a process of gradual familiarisation. Novice users frequently sought support from partners and family in the initial stages, becoming more independent as they gained confidence in their ability to use the system. Familiarisation could take anything from a couple of days to several months depending on participants’ disposition, aptitude and personal circumstances. Some novice users were actually surprised at how easy they found it to use the system: ‘it’s so simple to do that it’s no problem whatsoever, I don’t find it a problem’ (P13).

This gradual but relatively seamless adoption of the system contrasted with the experience of two participants who opted to withdraw from the study within a few days of consent. A woman with an underlying anxiety disorder became so worried using the system on her own that she could not bear to be in the same room as the equipment. The second early withdrawal involved a participant with advanced HF who found using the system too burdensome. Related to this experience, another participant gave up trying to learn how to use the tablet computer after a short period because he found it too complicated. This did not however deter him from using the system measurement devices for self-monitoring purposes as requested by his GP and HF nurse. This suggests that certain moderators of use merit consideration in future large-scale application of health technology for HF management. These are underlying physical and mental barriers, and reluctance to engage with digital technologies.

By the end of the study, eight out of the 27 novice users needed ongoing support in the form of physical help, reminders, and technological guidance. All but one of these cases represented couples, who had developed routines in which the non-participant would either operate the tablet computer or help the participant read the questions or instructions in case of eyesight problems, or assist with the use of the measurement devices (e.g. putting on the blood pressure cuff).

Participants’ levels of interaction with the system were also influenced by their digital technology proficiency, with novice users being less likely to review their daily readings or look at the ‘how to keep healthy’ pages. These users described how they learnt the basic monitoring tasks and were worried about doing anything else either for fear of damaging the system or getting lost: ‘I haven’t got a clue about anything beyond that – it is a different world’ (P3), ‘Well the parts that I’m used to are sort of routine if anything sort of strange happens, different, and I’m lost’ (P2). These experiences suggested that the system needed to contain additional support functions, such as written and animated guidance and an inbuilt means of accessing technical support. The latter was included in the course of the study.

### Establishing a routine

Over the course of the study, set patterns of use emerged, with most participants incorporating the use of the system into an established daily routine. For example, one woman kept the system in her kitchen and used it while she was making an early morning cup or tea, and another man would go to his office after breakfast to complete his measurements. Others used the system before taking their morning medications and a few participants used the system before going to bed. Establishing a routine was viewed as an important cue: ‘I find if I incorporate it into a routine I remember it’ (P23).

Others preferred to use the system less frequently, every couple of days or once a week. Either because they thought that daily use was excessive, or that they felt their symptoms were well controlled. ‘But there again I don’t believe in doing that every day. You’d just start, you’d get, you’d turn yourself into a hypochondriac’ (P10). Interestingly some of these participants also described how they would use the system when they felt unwell as a check to see if they needed to seek medical attention. In this way, the system offered a means of intermittent self-assessment. Three participants (P10, P12, P15) also related how they gradually lost interest in self-monitoring because their readings were stable and nothing ever changed. ‘I was so constant all the time, so I thought what is the point, if it was varying things were happening I would have continued, but it was so constant’ (P12).

Regular use of the system was also influenced by participants’ interactions with health professionals; one man attended a pain clinic daily and did not see the need to repeat physiological observations, and another participant ceased self-monitoring during a period of intense follow-up by a specialist nurses and doctors. Conversely, several participants recounted how their daily use of the system helped their GPs to monitor their blood pressure and titrate their medications.

### Usefulness

This section explores participants’ views on the usefulness of the system at the stage when related self-monitoring activities have become integral to their daily lives. Essentially this section expands on objectification by looking more closely at what occurs after users have become more familiar with the system.

### The practice of self-monitoring

Only four participants had previously been involved in self-monitoring of health related measures. One of these suffers from a rare immune condition and records data to share with her health-care providers when relevant. The other three keep detailed records in online applications, in Excel spreadsheets or paper notebooks but do not actively share these with health professionals. Self-monitoring was defined as ‘keeping account of myself’ (P9) by one of these participants, a way of knowing more about one’s health and learning more about specific readings such as blood pressure and weight.

As self-monitoring was a novel activity for many participants, they were often unclear about how to evaluate the data they were collecting. Whilst most participants were keen to record their symptoms and measurements and increase their understanding of their condition, they were concerned and hesitant about assuming responsibility for interpretation. Indeed, this perceived responsibility was described as worrying by one couple (P3) because they did not know whether the participant’s measurements were within acceptable parameters. Similarly, three other participants (P13, P36, and P38) stated that they preferred to leave the interpretation of their measurements to health professionals. Despite some concerns about interpretation, the practice of self-monitoring was described variously as ‘reassuring something that increases your confidence, helps you set and evaluate exercise and weight loss goals, and enables you to gauge your need for medical attention’.

Self-monitoring also played a role in medical review and medication titration. Of their own volition, several participants took the tablet computer with them to medical appointments in order to show their doctor or nurse their readings. Two participants (P30, P20) also relayed how they used their readings to prevent fluid retention by adjusting their diuretics dose in accordance with changes in their weight.

### Communication and integrated resources

The system included two communication functions, individual messaging from the research team to the participant and a ‘contact me’ button. The former was primarily used to provide feedback on participant’s readings, for example to advise them to seek medical attention, and the latter was mainly used to inform the team about any problems the participant was experiencing in using the system. Participants described receiving a message from the team as highly motivational since it provided evidence of a connection between themselves and health professionals. They were not just monitoring their symptoms in isolation but there was someone else reviewing their data with them. The contact button in turn served a dual purpose; it helped resolve technical problems, and humanised the system by creating opportunities for connection and conversation.

The system included a ‘how to keep healthy’ section where participants could access video clips. These included animations illustrating what happens when you have HF, and a collection of patients' stories about their experiences of living with HF.^27^ Participants expressed varied opinions about these resources, ranging from very positive to ambivalent. A recently diagnosed man found that the patients' stories helped him come to terms with his condition whereas others were not interested in hearing about others peoples’ experiences. For some this was because they thought they knew all they wanted to know about their condition, while for others it was because they were more interested in materials tailored to them individually.

### Conversion and aspirations

In this section, we explored the idea of conversion, the stage when a product gains a ‘taken for granted’ status i.e. when use of the system becomes an intrinsic part of users’ lives in the same way as brushing their teeth. In the context of research, this is not straightforward since an intervention is usually applied within a specified time period. However, since this usability study is part of a longer-term research programme, we were able to ask participants if they would like to continue using the system before the next study started. This was a voluntary option and they were under no obligation to participate in the subsequent study if they opted to continue using the system. Out of the 48 participants who completed a final exit visit, 20 independently chose to continue using the system for several months. At the exit visit these participants were also asked if they would use the system if it was available as part of routine care, and if they would recommend it to other patients. All but three participants stated they would use it, and all but one would recommend it to others.

In relation to the question of ‘conversion’, several participants shared their aspirations for ongoing developments of the system. Two participants (P6, P15) were keen for the system to help with the review of their medications and another participant (P44) suggested that the system should include different levels of sophistication (basic, intermediate, advanced) depending on individual requirements. This participant also suggested that clinical staff reviewing the monitoring data should send weekly communiqués to patients containing advice and reassurance. Participants with other co-morbidities wanted to be able to include blood glucose and peak flow readings to their regular monitoring activities, and another participant wanted more information on medications in general and anticoagulants specifically. Others talked about the need to include additional resources such as weight loss programmes, exercise plans, information about different types of HF and links to relevant organisations and local groups. Finally, to help patients deal with the uncertainties of living with HF it was suggested that the system should include a section on helping patients prepare for medical consultations by equipping them to ask pertinent questions about prognosis and quality of life.

## Discussion

### Principal results

Initially altruism and a desire to give something back to the health-care system were the dominant motivational factors that participants cited for agreeing to be involved in the development of the mhealth system. These sentiments outweighed hesitations expressed by some participants about their lack of technical competence. Whilst altruistic motivations continued to characterise participants’ interactions with the mhealth system, the benefits of technology enabled self-monitoring became more apparent to them as they gained confidence in operating the system and incorporated its’ use within a regular routine. Although participants had been told that the development of the system was complementary to their routine care, many began to view the system as a ‘safety net’ and an extension of care that helped them to feel connected, be more proactive and gain more control over their condition. This was not however the case for all and in a small group of participants we observed several reasons for non-engagement. Declining interest in self-monitoring due to stable readings was the predominant reason for withdrawal or less frequent use of the system, and pre-existing mental and physical disabilities prevented two participants from using the system.

Participants’ speed of familiarisation and level of interaction with the system was influenced by their digital proficiency. Novice users were less likely to check their measurements on the graphs provided and tended to be more apprehensive about assuming responsibility for interpreting their readings. The primary value of the system for novice users was the reassurance gained from the link to external medical review. This connection was generally highly prized and used by some as a platform for self-assessment, a means of gauging their need for medical intervention and setting and evaluating personalised health goals (e.g. daily exercise).

### Comparison with prior work

Like others, we have demonstrated that patients of different ages and technological abilities can adopt technology enabled home monitoring systems relatively easily.^28–30^ From this literature it is apparent that patients with chronic conditions value the continuity of care and legitimised contact with health practitioners such systems can offer, our experience also indicates that technology enabled home monitoring can help patients gain more control over their condition and set personalised self-care goals. Similarly Seto et al.^11^ document how real-time feedback enhances self-care by providing patients with timely instructions and support, and argue that harnessing the value of these ‘teachable moments’ is critical in telemonitoring. This evidence suggests that rather than undermining positive ageing and self-reliance,^13^ well-designed and supported telemonitoring systems can improve patients’ experience of living with HF. The majority of our participants were keen to access telemonitoring as part of routine care and 20 people opted to continue to use the mhealth system for several months after the study. However, we accept that users with higher levels of digital competency were more likely to gain self-care benefits from using the SUPPORT HF mhealth system.

Gaps in evidence exist about the required duration and frequency of use of telemonitoring systems in order to develop self-competency skills and confer lasting health benefits. For example, Agboola et al.^31^ note that lower hospitalisation and mortality rates achieved during an intervention group’s involvement in a four-month ‘Connected cardiac care programme’, did not persist in the subsequent eight months. Given that increasing the duration of such programmes for all patients may not be cost-effective, they highlight the need to develop ways of stratifying HF patents according to risk and potential benefit, in order to determine and evaluate the optimal duration for home monitoring and the effect of less intensive, long-term home monitoring for different groups of patients.

Our results correspond with observations made by Hunich et al.^32^ about how digitally enabled self-monitoring can produce a sense of security when readings provide grounds for explaining symptoms and widen the scope of possibilities for taking action. On the other hand, readings can also be experienced as depressing, worrisome and, at times, disturbing. Hence, we would argue that developers of digital health systems need to pay more attention to reducing any anxieties which self-monitoring can evoke, and to helping users, including their caregivers, understand and act on their readings. This is not so that patients just become more compliant with medical instructions or learn to manage their condition in an almost professional manner, but so that they are empowered to make informed and autonomous choices about what they need to do to improve or maintain their health.^33^ Herein lies the definitive goal of user involvement namely the development of stratified and adaptive digital systems that reflect patients’ priorities and promote collaborative clinical follow-up.

### Strengths and limitations

Despite our study limitations: limited sample size, selection bias during recruitment and the possibility that the application of theory may have added a deductive angle to our analysis, we think we have made a good start at realising the goal of developing a system that reflects patients’ priorities. By involving users in the development of a mhealth system we were able to learn from their interactions with self-monitoring equipment and refine the system accordingly. It was evident that patterns of engagement with the mhealth system changed over time and varied according to patients’ need and capacity to use the system. Novice technology users were capable of adopting the technology but their requirements were different, in terms of both system design and time required for learning the system. Future systems will need to become more personalised and adaptive if people with wide-ranging and changing capacities and needs are to be included. Our work demonstrates the importance of investing time into understanding how different types of users adopt and incorporate digital-health technology into their daily lives. Accounting for the diversity of user experiences in the development and maintenance of mhealth systems is likely to increase the extent of successful uptake and impacts on outcomes for patients and providers.

## Conclusions

We achieved our aim of developing a user-friendly home monitoring system with the majority of participants stated they would use it if it were available as part of routine care. Important concepts emerged that are useful for the future development of mhealth technologies: (a) connection: a key motivator was the sense of connection the system provided to the study team, outweighing the ability to view daily readings or health information; (b) routine: continuous usage was strongly associated with patients fitting the monitoring into their daily routine; and finally (c) personalisation: participants were able to use the system with different levels of involvement and understanding, some patients taking active control of their health whereas others appreciated using the system more passively. We found that this often correlated with their level of digital competency and that further personalisation of the system was desirable to patients.

These findings are now being incorporated in the second phase of the study, SUPPORT-HF 2, to make the system more personal by incorporating individual preferences or providing health information that is tailored to each individual’s diagnosis, medications or co-morbidities, and more interactive by facilitating two-way communication between patients and study team.
